# Microfluidic device for electromembrane extraction with a micro-pillar stabilized liquid membrane

**DOI:** 10.1007/s00216-026-06427-z

**Published:** 2026-03-06

**Authors:** Anna Thu Hoai Nguyen, Nickolaj J. Petersen, Stig Pedersen-Bjergaard, Jörg P. Kutter

**Affiliations:** 1 Department of Pharmacy, Faculty of Health and Medical Sciences, University of Copenhagen, Universitetsparken 2, Copenhagen, 2100 Denmark; 2 Department of Pharmacy, Faculty of Mathematics and Natural Sciences, University of Oslo, P.O Box 1068, Blindern, 0316 Oslo Norway

**Keywords:** Sample preparation, Microextraction, Microfluidic device, Stabilized interfaces, Electromembrane extraction

## Abstract

Electromembrane extraction (EME) is a microextraction technique where charged analytes are transferred across a liquid membrane, under the influence of an electrical field, and enables selective and efficient sample cleanup. In this paper, we present for the first time a microfluidic device for electromembrane extraction where the liquid membrane was held in place by rows of micro-pillars. This design allows rapid membrane replacement, low-voltage extractions, and extended reuse, addressing key limitations of conventional EME formats. The microchip was fabricated in a stoichiometric thiol-ene polymer, offering a balance between hydrophobic and hydrophilic properties and supporting controlled flow of both aqueous and organic phases. Lidocaine was selected as the test substance for the experiments. Extraction parameters were optimized, and with a sample flow of 0.5 µL min^−1^, acceptor flow of 2.0 µL min^−1^, 0.1 µL 2-nitrophenyl octyl ether (NPOE) as the liquid membrane, and a 10-min extraction time, recoveries up to 50% were obtained. Linearity was demonstrated from 5 to 20 µg mL^−1^ (*R*^2^≈0.99), repeatability was acceptable (16.0% RSD), and carryover was minimal (1.3%). The system remained stable for at least 60 min of continuous extraction, and the liquid membrane could easily be replenished without chip disassembly. The miniaturized design and low solvent consumption yielded a high AGREEprep greenness score of 0.79, confirming the greenness and sustainability of the method. This work is a first step towards a versatile, reusable, and sustainable EME microchip and represents a key step towards a highly flexible device with potential for on-site analytical applications.

**Graphical abstract:**

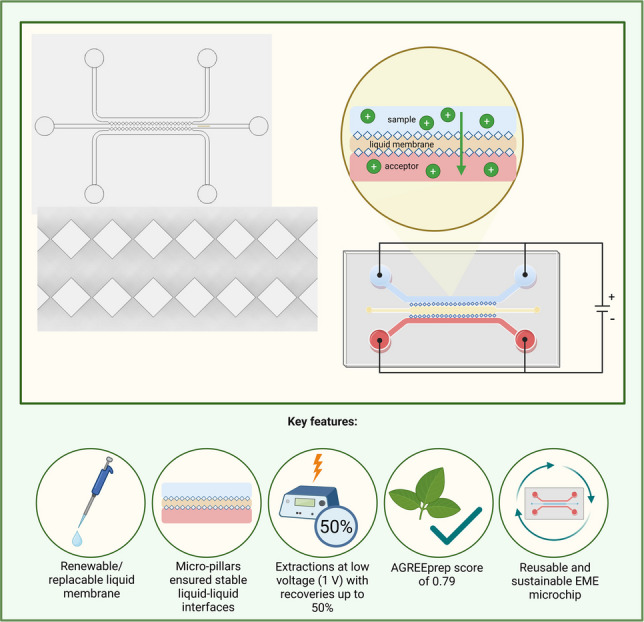

**Supplementary Information:**

The online version contains supplementary material available at 10.1007/s00216-026-06427-z.

## Introduction

Electromembrane extraction is a microextraction technique, where target compounds are extracted from a sample solution, across an organic liquid membrane and into an aqueous acceptor solution. Mass transfer and extraction are facilitated by an electric field sustained across the organic phase, providing efficient preconcentration and sample cleanup from complex aqueous samples [1]. In most implementations of EME, the liquid membrane is immobilized within a porous polymer and is thus commonly referred to as “supported liquid membrane” (SLM). Since its introduction in 2006 [2], EME has been widely applied to the extraction of drug substances from biological fluids (blood, urine, and saliva), as well as environmental and pharmaceutical applications. The technique provides a unique and tunable selectivity based on the electric field, the composition of the SLM, and pH, and provides a green and sustainable alternative for the extraction of acids, bases, and permanently ionic analytes [1, 3]. Because the acceptor normally is aqueous, the method is directly compatible with LC and CE [4].

Traditional EME setups make use of hollow fibers [2], where a porous and hydrophobic hollow fiber, typically made of polypropylene, is used to support the organic solvent. Numerous setups have been presented, including 96-well systems and a commercially available setup based on the use of conductive vials. In this latter setup, an EME cell comprises two conductive vials, a flat circular support membrane impregnated with membrane solvent, and a union holding the SLM between the two vials [5, 6].

Downscaling and implementation of EME into microfluidic devices offer the potential of integrating sample preparation, separation, and detection into a single device [6]. Previous microfluidic devices used SLMs, where the organic phase was immobilized in a porous polymeric membrane [1, 4, 6–8]. These membranes were often sandwiched between several layers, raising challenges with respect to ease of fabrication, leakages due to bonding issues, and loss of analyte in the organic phase due to diffusion in the membrane layer, which all can contribute to issues with carryover and poor repeatability [4, 7]. Furthermore, replacing such membranes can be cumbersome or even impossible, strongly limiting the versatility and reusability of such chips.

An alternative to SLMs is free liquid membranes (FLMs), where the organic phase is suspended between two aqueous phases without the need for a porous supporting material [9]. This approach simplifies the membrane preparation and avoids certain limitations of SLMs, as mentioned above, while offering potential for improved mass transfer, reduced analyte loss, easier membrane renewal, and reduced carryover between extractions [3, 9–12]. In 2014, Kuban et al. [9] presented the technique micro-electromembrane extraction (µEME) using electrically induced transfer of analytes across FLMs. Here, a disposable extraction unit was proposed and made of a short segment of transparent perfluoroalkoxy tubing filled with three liquid plugs serving as acceptor solution, FLM, and donor solution, respectively [9]. With this setup, charged dyes were extracted with recoveries above 60% and with %RSDs below 5%. Since then, extraction from undiluted biological samples, the use of volatile FLMs, simultaneous extraction of anions and cations, and the use of multiple FLMs and acceptor solutions have been reported [13–16], as well as optimizing parameters for the extraction process. Optimized parameters include composition and volume of donor/acceptor solution, applied potential, thickness of FLM, and extraction time [13].

However, the stability of FLMs can be compromised by the electrical potential, pressure fluctuations, and flow conditions. A closer look at the physics underlying the stability of aqueous-organic liquid interfaces and co-flows (also in microchannels) was undertaken by Berthier et al. [17], who combined theory, simulation, and experiments in their study. A particularly interesting case was the exploration of how two immiscible liquids can flow side by side in a microchannel separated by vertical rows of micro-pillars, maintaining interface stability and allowing mass transfer across the interface by diffusion [17]. They modeled the stability of interfaces defined by the pillars and the potential limitations of the efficiency of the mass transfer. Nevertheless, this study provides inspiration and design guidance on how the use of micro-pillar arrays could enhance FLM stability by carefully considering all aspects of the channel geometries and the pillar geometries, including size, shape, and spacing [17]. A very essential aspect in all this is the contact angle between the liquids and solids involved, and therefore also the material of the chip/pillars.

Material selection plays a crucial role in the fabrication of reliable microfluidic devices for EME. Thiol-ene polymers have emerged as highly versatile materials for lab-on-chip applications due to their favorable properties, including low shrinkage during polymerization, high optical transparency, high chemical resistance, and biocompatibility [18]. These characteristics are advantageous in EME, where the microchip must withstand exposure to organic solvents [19] and applied extraction potentials for extended periods of time. Additionally, the fabrication of microchips using thiol-ene materials is low cost, flexible, and rapid, without the need for surface activation or treatment prior to bonding [18]. In 2011, off-stoichiometric thiol-ene (OSTE) was introduced by Carlborg et al. [20], opening for even more versatile applications. With this approach, the monomers are used in nonstoichiometric ratios, resulting in an excess of either thiol or allyl functional groups on the surface after polymerization and bonding. These residual functional groups can participate in click chemistry reactions, allowing for straightforward and selective surface modification or functionalization. OSTE materials enable tailored surface properties, such as controlled wettability, or the immobilization of functional molecules for applications in biosensing, enzymatic reactions, or separations on-chip [18, 20]. The many advantages of thiol-ene polymers make the material versatile and suitable for a broad range of lab-on-chip applications. Moreover, the potential to implement greener, more sustainable approaches during fabrication and use makes the polymers attractive for future developments in microfluidics and analytical chemistry.

In this work, we present the initial development of a new, thiol-ene polymer-based microfluidic chip for EME, where the liquid membrane was stabilized in a central channel by rows of micro-pillars. With this new arrangement, the liquid membrane was easily replenished, and the microchip was cleaned and reused for a large number of extractions. We discuss the initial design and fabrication of this highly flexible microfluidic chip, along with the first set of proof-of-principle experiments. Technical aspects were highlighted in this paper, while extraction from real samples and applications will be covered in forthcoming research.

## Experimental

### Chemicals and solutions

Pentaerythritol tetrakis(3-mercaptopropionate), 1,3,5-Triallyl-1,3,5,triazine-2,4,6(1H,3H,5H)-trione, nitrophenyl octyl ether (NPOE), sodium hydroxide, pethidine hydrochloride, nortriptyline hydrochloride, methadone hydrochloride, haloperidol, and loperamide hydrochloride were purchased from Sigma-Aldrich (St. Louis, MO). Phosphoric acid 1 mol L^−1^ and hydrochloric acid were purchased from Merck (Darmstadt, Germany). Lidocaine hydrochloride was purchased from Mecobenzon (Copenhagen, Denmark). Ethanol was purchased from VWR (Rosny-sous-Bois, France). A Sylgard 184-poly-(dimethylsiloxane) (PDMS) elastomer kit was purchased from Dow Corning (Midland, MI). Deionized (DI) water with resistivity higher than 18 MΩcm was used from a remote water purification system (Direct-Q® 3 UV, Merck, Darmstadt, Germany). Polylactic acid (PLA) filament for 3D printing, 1.75 mm, was purchased from PriGo (Odder, Denmark).

A stock solution of lidocaine 1 mg mL^−1^ was prepared in ethanol and stored at 4 °C. From this, a test solution was prepared by dilution with 10 mM hydrochloric acid (HCl) to a final concentration of 20 µg mL^−1^. In addition, a mixed stock solution containing 1 mg mL^−1^ of haloperidol, lidocaine, loperamide, methadone, nortriptyline, and pethidine was prepared in ethanol. Sample solutions containing the six compounds were prepared by dilution of this stock solution with 10 mM HCl. For all experiments, 10 mM HCl was used as the acceptor solution.

### Fabrication of the microfluidic chip

**Fig. 1:**
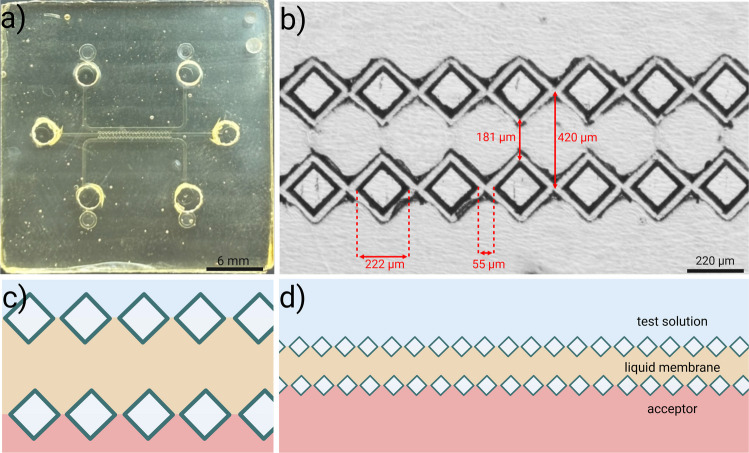
Design and details of the microfluidic chip for EME. **a** Photograph of the fabricated thiol-ene microchip showing the overall three-channel layout and inlet/outlet reservoirs. **b** Microscope image of the diamond-shaped micro-pillar arrays separating the three channels. The micro-pillars form two parallel rows that confine the liquid membrane in the middle channel while leaving open gaps between adjacent pillars, allowing direct contact between the liquid membrane and the adjacent aqueous phases. The spacing between pillars is approximately 55 µm, each pillar is 222 µm across diagonally, and the distance between the two rows is 181–420 µm, corresponding to the width of the liquid membrane. **c** Schematic cross-sectional illustration showing how the liquid membrane occupies the middle channel and is stabilized by the micro-pillars, while the test and acceptor solution flow in the outer channels. **d** Schematic overview of the three-phase configuration, illustrating the test solution, liquid membrane, and acceptor solution in direct contact through the gaps between pillars, enabling mass transfer across the well-defined liquid–liquid interfaces. Created in BioRender. Nguyen, A. (2026) https://BioRender.com/2lh503o

The microfluidic chip is illustrated in Fig. 1. It consisted of two layers: a bottom layer featuring two outer channels for the test and acceptor solutions, respectively, and a middle channel for the liquid membrane, stabilized by a row of micro-pillars at each interface; and an upper layer that serves as a lid.

The thiol-ene microchip was fabricated using a two-step replica molding process, as described in more detail in [18]. Briefly, master molds were designed using a computer-aided design (CAD) software (Autodesk Inventor Professional 2024, San Francisco, CA) and micro-milled (Minitech 3, Minitech Machinery Corporation, Norcross, GA) into poly(methylmethacrylate) (PMMA) plates (100 mm × 100 mm × 5 mm, NordiskPlast, Randers, Denmark). These PMMA molds were then used to make polydimethylsiloxane (PDMS) molds by pouring PDMS mixture (Sylgard 184-poly-(dimethylsiloxane)) into the master molds and curing for 2 h at 80 °C. A stoichiometric thiol-ene formulation was prepared by weighing and mixing thiol- (pentaerythritol tetrakis(3-mercaptopropionate)) and ene-monomers (1,3,5-triallyl-1,3,5,triazine-2,4,6(1H,3H,5H)-trione) in a 1:1 molar ratio, followed by degassing under vacuum to remove air bubbles.

Both layers of the microchip were fabricated by pouring the thiol-ene mixture into the respective PDMS molds, covered with a flat PDMS sheet, and cured under UV-light (85 mW cm^−2^ at 365 nm, Dymax EC 5000 Series UV curing flood lamp, Dymax Corp, Torrington, CT) for 6 s on each side. For the upper layer, six holes were punched out for the channel outlets and inlets using a 2.0 mm biopsy punch (Medblades.com, Addison, IL). The two layers were laminated using a roller to ensure alignment and eliminate any air bubbles, followed by bonding under UV-light for 2.5 min on each side. After bonding, excess thiol-ene was trimmed with scissors, yielding a final chip size of 25 × 25 × 2 mm. Finally, the microchip was heat-treated in an oven at 200 °C for 20 h (VWR® VENTI-Line, VWR International BVBA, Leuven, Belgium), as described in [19].

#### Chip dimensions

The microchip comprises three parallel microchannels: a channel for the sample (often also denoted “donor” in the context of EME), a channel for the acceptor, and a middle channel for the liquid membrane. The channels are separated by square diamond-shaped micro-pillar rows, allowing mass transfer from one phase to another while maintaining a stable three-phase system. The channel lengths for extraction are 6.4 mm, and the width of the outer channels is 300 µm, while it is 181 µm for the middle channel. The pillars are each 222 µm across diagonally with a corner-to-corner distance of roughly 55 µm after milling. Smaller inter-pillar spacings are possible, with either smaller drill bits or a different mill bit routing. Smaller bits are more prone to mechanical failure, while a more advanced routing requires careful editing of the instruction files fed to the milling machine. Thus, by designing the spacing to be very small (5 µm) in the CAD drawing and using a 50-µm end mill, the final distance came out to 55 µm. Around the micro-pillars is a raised 35-µm edge (see Fig. 1d) relative to the depth of the main channels, which is 100 µm. This was found beneficial in maintaining stable liquid–liquid interfaces.

### Electromembrane extraction on chip

A custom chip holder was designed using the same CAD software as mentioned earlier, and 3D printed in PLA (Prusa MK4S, Prusa Research, Prague, Czech Republic). The holder allowed secure placement of the microchip and ensured proper alignment of inlet and outlet ports with electrodes and connecting Tygon tubing.

The EME procedure was initiated by loading the test and acceptor solutions into two 500-µL glass syringes (Gastight # 1750 syringe, Hamilton, Bonaduz, Switzerland), which were connected to the chip with Tygon tubing (Tygon LMT-55, i.d. 1.02 mm, MIKROLAB-FRISENETTE, Viby J, Denmark) that were, in turn, connected to inlet electrodes (1/16 stainless steel HPLC tubing, i.d. 600 µm). Stainless steel tubing segments served as inlet and outlet electrodes and were positioned directly at each channel inlet and outlet. The glass syringes were attached to two syringe pumps (Cole-Parmer Single-Syringe Infusion Pump 74900 Series, Cole-Parmer, Illinois, USA) for controlling the flow rates. The chip was then placed in the 3D-printed holder and secured using a laser-cut PMMA lid of 5 mm thickness and screws. Once the chip was secured, flow was initiated through both channels with the syringe pumps and the solutions filled the sample and acceptor microchannels. The liquid membrane, NPOE, was introduced into the middle channel by pipetting into the inlet tubing, and the channel was filled by pressure applied from the pipette. The flow was visually monitored until the liquids reached the sample and acceptor outlet, where additional Tygon tubing was connected to outlet electrodes. An extraction potential, typically 1 or 2 V, was applied using an external power supply (BK9206B Power Supply, B&K Precision Corporation, CA, US), with the anode connected to the sample inlet and outlet electrodes, and the cathode connected to the acceptor inlet and outlet electrodes, thereby establishing an electric field across the liquid membrane. The system was primed by flushing for 10–20 min prior to extraction. During extraction, a constant potential was applied, and the electrical current was recorded using a multimeter (Fluke 289 multimeter, Fluke Corporation, Washington, USA) connected to a computer for data logging. During extraction, 20 µL aliquots were collected from the acceptor outlet using a pipette and transferred into CE vials and analyzed with CE-UV.

### Capillary electrophoresis

Capillary electrophoresis (CE) was performed with an Agilent 3D CE instrument (Agilent, Palo Alto, CA), operating at 200 nm for detection. The running buffer was 25 mM sodium dihydrogen phosphate adjusted to pH 2.7 with ortho-phosphoric acid. Injections were done at 40 mbar for 11 s, and separations were performed at 25 kV in a 50-µm-I.D. fused-silica extended light path capillary (Agilent, Santa Clara, CA) with an effective length of 43.7 cm.

### Data analysis and calculation of recovery

All data analysis was performed in Agilent OpenLAB.

Extraction recovery for lidocaine was determined using a calibration curve constructed from standard solutions in the concentrations of 1, 2, 5, 10, 15, and 20 µg mL^−1^ of lidocaine in 10 mM HCl. The calibration curve was based on the ratio of the peak area to the migration time, which accounted for variations in separation times. A new calibration curve was constructed every week to account for long-term fluctuations in the CE system.

The recovery $({R}_{i})$ of lidocaine $(i)$ was calculated according to Eq. 1:

1 $${R}_{i}=\frac{{n}_{{a}_{i}}final}{{n}_{{s}_{i}}initial}=\frac{{V}_{a}*{C}_{{a}_{i }}final}{{V}_{s}*{C}_{{s}_{i}}initial}*100\%$$

Here, ${n}_{{a}_{i}}final$ is the amount of lidocaine $\left(i\right)$ extracted into the acceptor solution, and ${n}_{{s}_{i}}initial$ is the amount of lidocaine $(i)$ originally present in the processed test volume ${V}_{s}$. ${V}_{a}$ is the volume of the acceptor phase processed through the chip with a final concentration ${C}_{{a}_{i }}final$, and ${C}_{{s}_{i}}initial$ is the initial concentration of lidocaine in the test solution.

## Results and discussion

### Proof of principle

**Fig. 2:**
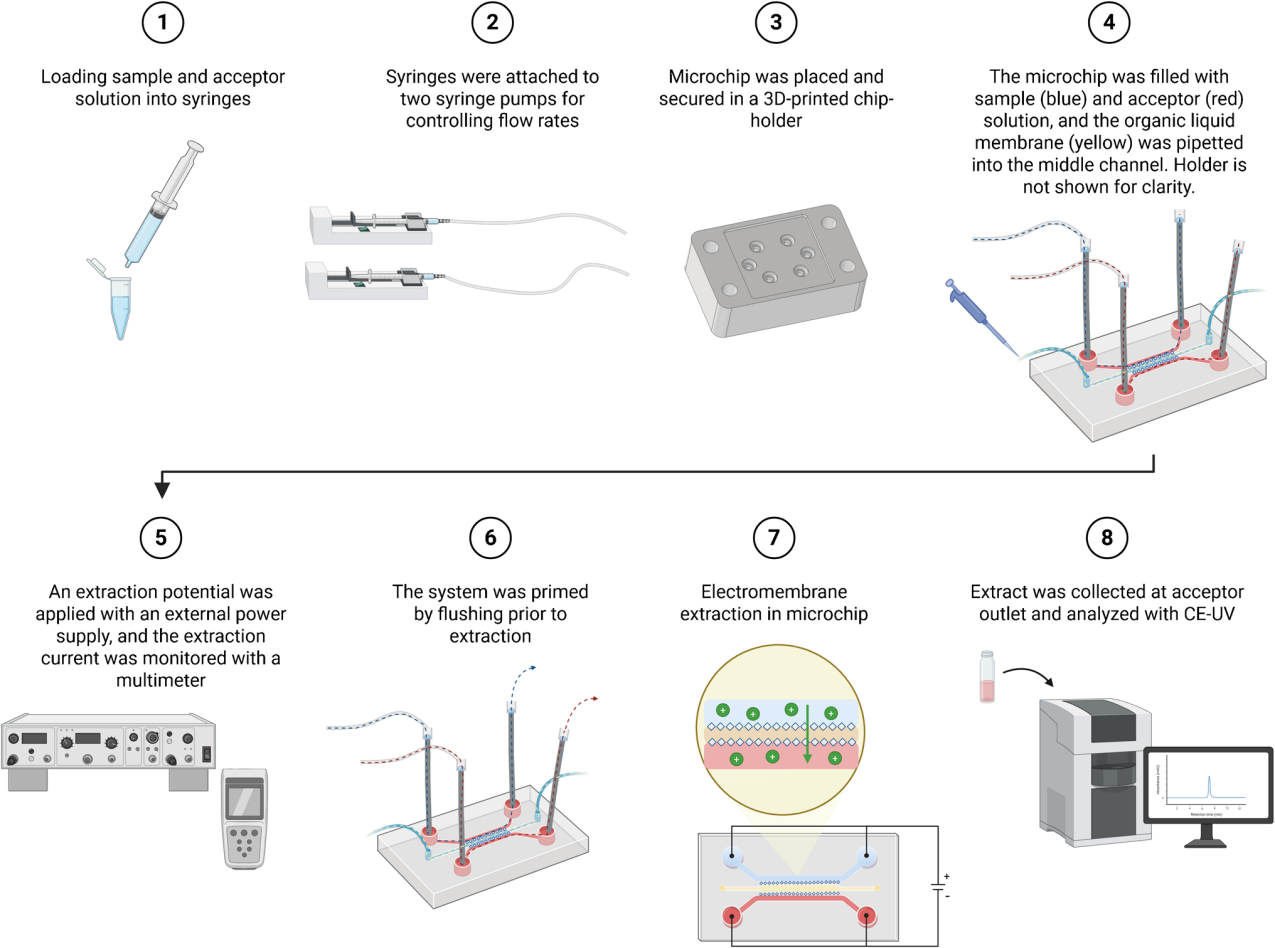
Schematic overview of the EME procedure on a microchip. The process includes the following steps: (1) test and acceptor solutions are loaded into two separate syringes; (2) the syringes are connected to syringe pumps for controlling flow rates; (3) the microchip is secured within a custom-designed 3D-printed holder; (4) test solution (blue) and acceptor (red) solutions are introduced into their respective channels, while the liquid membrane (yellow) is pipetted into the middle channel; (5) an extraction potential is applied using an external power supply, and the current is continuously monitored; (6) the chip-system is flushed to prime the channels prior to extraction; (7) analytes are extracted from the sample channel, across the liquid membrane, and into the acceptor channel under the electric field; and (8) the acceptor extract is collected and subsequently analyzed by CE-UV. Created in BioRender. Nguyen, A. (2026) https://BioRender.com/q4u3e44

The principle of the EME chip and the overall workflow are illustrated in Fig. 2. A micro-syringe pump was used to introduce the test solution into the sample channel at a flow rate of 0.5–2.0 µL min^−1^. Another micro-syringe pump was used to introduce the acceptor into the acceptor channel. The acceptor was 10 mM hydrochloric acid at a flow of 0.5–2.0 µL min^−1^. 2-Nitrophenyl octyl ether (NPOE) was used as a liquid membrane and was selected based on its well-established performance in EME systems [21] and its suitability for bases in the log P range of 2–6. NPOE was pipetted in excess (10 µL) into the middle channel of the chip. The latter was separated from the sample and acceptor channels by micro-pillars, as illustrated in Fig. 1. About 0.1 µL of NPOE served as the effective liquid membrane. The test solution was lidocaine dissolved in 10 mM hydrochloric acid (20 µg mL^−1^). Lidocaine was selected as the test substance. Lidocaine has been extracted with high efficiency with NPOE in traditional EME systems and was considered an appropriate test substance in the present work [22].

The extraction was initiated by application of an electrical field across the liquid membrane. The anode (positive electrode) was inserted into the sample channel, and the cathode (negative electrode) was inserted into the acceptor channel. In the first experiments, the extraction potential was set to 2.0 V, and extraction was performed for 10 min. During this period, 20 µL of test solution was pumped through the sample channel, and lidocaine was extracted through the liquid membrane and into the acceptor solution. The total volume of acceptor solution collected was 20 µL, and the concentration of lidocaine was measured with CE-UV (typical CE electropherograms of the donor solution before extraction and the acceptor solution after extraction, respectively, are shown in Fig. SI1).

### Chip material and design

One consequence of miniaturization is the increased surface area-to-volume ratio, and it thus becomes increasingly important to control the surface properties of materials for chip fabrication. For the intended purposes here, the surface wettability plays a crucial role in determining flow properties, as it directly affects flow behavior and the stability of the liquid interfaces [18]. The microfluidic chip in the presented work was fabricated from stoichiometric thiol-ene, a polymer known for its favorable surface properties and high mechanical strength in microfluidic applications [18, 23]. For stoichiometric thiol-ene in contact with water, the reported contact angle has been in the range of 60–80° [18], indicating that the material has both hydrophobic and hydrophilic properties. In the current experiments, the water contact angle was measured to 70°, and the NPOE contact angle was measured to 36°. With the selected material (thiol-ene) and the geometry of the micro-pillar structures, the aqueous sample and acceptor were loaded into their respective channels at 2.0 µL min^−1^ without permeation into the middle channel, after which the organic phase was introduced by pipette and, upon applying a slight pressure, entered the middle channel only. The combination of material properties and geometric restriction offered by the micro-pillars allowed reliable priming of both aqueous and organic phases and provided a stable interface.

Prior to use, the chips were heat-treated at 200 °C for 20 h to enhance their compatibility with organic solvents [19]. This reduces solvent-induced swelling and improves the chemical robustness of the chips. With chips stored for more than 3 to 4 weeks, however, the aqueous phases often merged slowly into the middle channel during the initial filling and extraction, causing loss of interface stability and displacement of the liquid membrane. Chips older than 4 weeks were therefore generally avoided. The dimensions of the channels, pillars, and inter-pillar distances in this particular design were chosen based on theoretical considerations [17] and the authors’ previous experience with thiol-ene-based microfluidic systems [18, 19].

### Membrane stability

The overall system stability was assessed by monitoring the duration for which the liquid membrane remained intact within the center channel. The stability was assessed by visual inspection and by recording the current throughout the extraction. A constant current indicated a stable system.

Flow rates were varied in the range of 0.2 to 5.0 µL min^−1^. In a first set of experiments, flow rates were equal in the sample and acceptor channels. As seen in Fig. SI2, at flow rates of 0.2 and 0.5 µL min^−1^, the current was constantly around 15 µA during 10 min of extraction at 15 V, indicating high stability of the liquid membrane and the corresponding interfaces. At flow rates of 1.0 and 2.0 µL min^−1^, the current was generally stable at 10–15 µA, but some variation was observed between chips from different production batches. Generally, each chip could be used for up to one month before stability was compromised. These chips showed slightly higher currents than freshly fabricated ones. Their associated current profiles were fluctuating due to small air bubbles entering the channels (Fig. SI2). At flow rates in the range of 3.0 to 5.0 µL min^−1^, the frequency and magnitude of the current fluctuations increased significantly, and the current often exceeded 30 µA. Such high currents are considered problematic in EME, as they may lead to electrolysis and related detrimental effects [24].

The flow rates between 0.2 and 2.0 µL min^−1^ were identified as the optimal range for maintaining a stable liquid membrane. Due to the small dimensions of the channels [17] and the sensitive confinement of the liquid membrane, higher flow rates disrupted or displaced the membrane or introduced air bubbles, compromising the system performance and stability [4].

In another set of experiments, the stability was investigated with different flow rates on each side of the liquid membrane. In one experiment, the test flow rate was constant at 2.0 µL min^−1^, and the acceptor flow rate was gradually reduced from 2.0 to 0.2 µL min^−1^. In a second experiment, the acceptor flow rate was constant at 2.0 µL min^−1^, and the test flow rate was gradually reduced from 2.0 to 0.2 µL min^−1^. In both experiments, the liquid membranes were stable based on the current measurements, as illustrated in Fig. SI2.

### Extraction potential

**Fig. 3:**
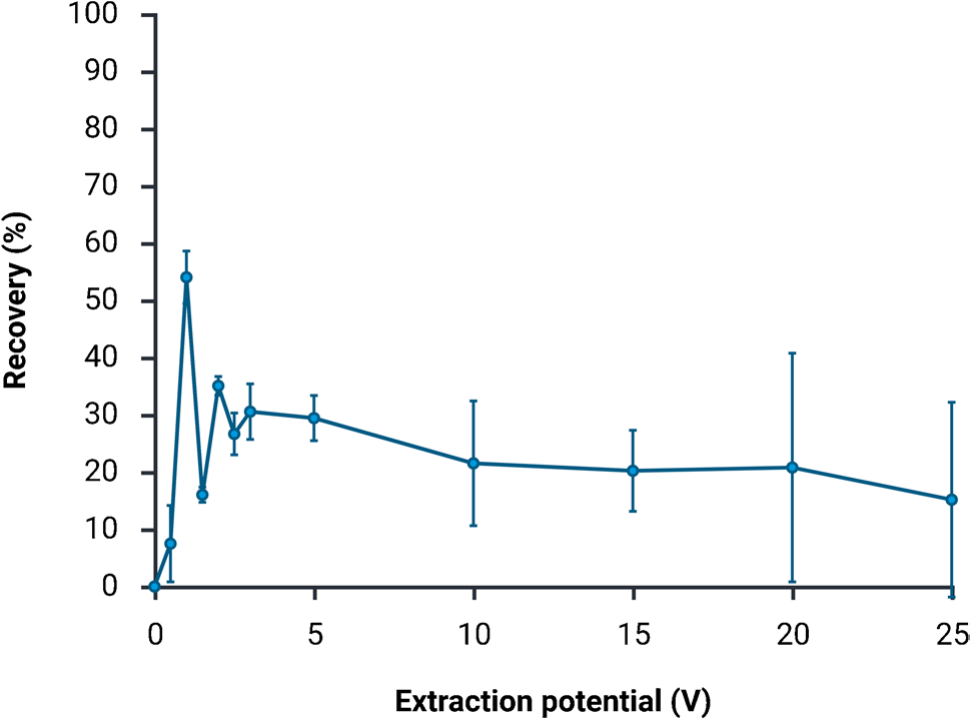
Extraction efficiency expressed as percentage recovery at different extraction potentials (0–25 V), using the microchip system (mean ± SD, *n* = 3). All extractions were performed at a flow rate of 2.0 µL min^−1^ in both sample and acceptor channels. Optimal extraction efficiency was achieved at low extraction potential (1.0 V), whereas the extraction efficiency declined at higher potentials

In a next set of experiments, the extraction potential was optimized (Fig. 3). With 0.0 V, no extraction occurred, i.e., no lidocaine was found in the acceptor solution. Consequently, it is reasonable to assume that there was also no mass transfer due to passive diffusion across the membrane. Recovery increased with increasing extraction potentials up to 1.0 V. At this extraction potential, the current was 0.1 µA. As the extraction potential was increased above 1.0 V, recovery decreased, and the current still increased linearly with voltage. The stability of the system decreased with increasing voltage, likely due to pH changes in the boundary layers (also discussed in “Flow rates”).

Compared to previously published microfluidic systems for EME operated at 15–300 V, the optimal extraction potential (1.0 V) was much lower in the current system [13, 23, 25, 26]. The low potential can be explained by several factors. Firstly, the liquid membrane was relatively thin (below 200 µm) and of low tortuosity, which reduced the electrical resistance and thus allowed for more efficient ion transport at lower voltages. Additionally, the overall channel dimensions and electrode distances were smaller, allowing for a more effective coupling of the extraction potential with the main potential drop occurring across the liquid membrane. Furthermore, both sample and acceptor were dynamic (i.e., flowing), facilitating continuous renewal of the sample and reduction of boundary layers, and thus, less voltage was required for compensation.

### Flow rates

Next, a series of experiments was conducted with various flow rates in the sample and acceptor channels. Sample and acceptor flow rates are key operational parameters, as the linear velocity of the aqueous solutions affects the mass transfer in and out of the liquid membrane [7, 27]. These experiments were performed to evaluate whether the flow rate influenced the extraction recoveries of lidocaine. The applied extraction potential was set to 1.0 V, and the sample and acceptor flow rates varied between 0.5 and 2.0 µL min^−1^. The extraction time was either 10, 20, or 40 min depending on the acceptor flow rate, in order to obtain a 20 µL extract for each condition.

**Fig. 4:**
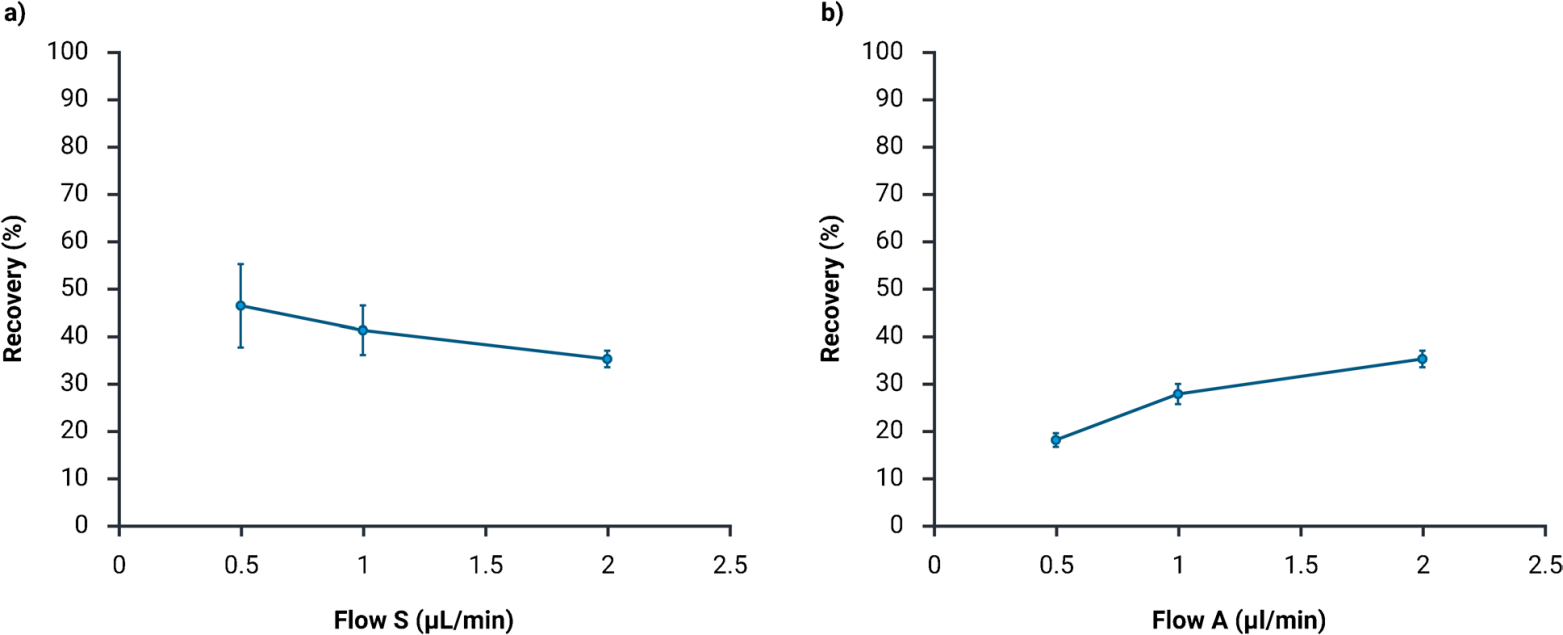
Extraction efficiency expressed as percentage recovery as a function of flow rate configurations (mean ± SD, *n* = 3). **a** Recovery as a function of different flow rates (0.5–2.0 µL min^−1^) in the sample (S) channel at 1.0 V. The flow rate in the acceptor (A) channel was constant at 2.0 µL min^−1^. **b** Recovery as a function of different flow rates (0.5–2.0 µL min^−1^) in the acceptor channel at 1.0 V. The flow rate in the sample channel was constant at 2.0 µL min^−1^

First, the flow rate in the acceptor solution was kept at 2.0 µL min^−1^, while the test flow was varied. Increasing test flow rate led to decreased recovery (Fig. 4a). With increasing flow rate, the residence time in the sample channel was reduced, and both electrokinetic and diffusive transport into the liquid membrane decreased. While a fully stagnant test solution would enhance recovery through prolonged residence time, the continuous acceptor flow would dilute the extract and make quantification difficult.

Next, the test flow rate was constant at 2.0 µL min^−1^, while the acceptor flow rate was changed. Increasing the flow rate led to increased recovery (Fig. 4b). With 0.5 µL min^−1^ acceptor flow, the residence time in the acceptor channel was 24 s, and the mass transfer across the liquid membrane/acceptor interface was likely limited by elevated pH in the acceptor/liquid membrane boundary layer [24]. When the flow was increased to 2.0 µL min^−1^, the residence time decreased to 6 s, the boundary layer decreased, and the mass transfer improved.

For the remaining experiments, the flow rate was set to 2.0 µL min^−1^ in both channels. Keeping the same flow rate on both sides of the liquid membrane reduced the mechanical pressure across the liquid membrane [17]. Furthermore, with 2.0 µL min^−1^, experiments were completed in 10 min, and the volume of the acceptor collected in the open experimental system was not affected by potential evaporation. With flow rates of 2.0 µL min^−1^ in each channel, no preconcentration was obtained.

### Extraction efficiency

With the test flow rate at 0.5 µL min^−1^, the acceptor flow rate at 2.0 µL min^−1^, and the extraction potential at 1.0 V, the recovery for lidocaine was approximately 50% as seen in Fig. 4a. We were unable to increase the extraction efficiency above this level, most probably due to the pillar structure. Further optimization of the pillar structure is a very resource- and time-demanding research and will be conducted and described in a forthcoming paper.

To further characterize the general applicability of the microchip system, five additional basic analytes were evaluated together with lidocaine. These included pethidine, haloperidol, nortriptyline, loperamide, and methadone. These compounds are in the log *P* range from 2.5 to 5.0 and have previously been used as model drugs in EME studies within the group. Extractions were performed using a mixture of all six analytes with sample and acceptor flow rates of 2.0 µL min^−1^ and an extraction potential of 1.0 V. The extraction recoveries are shown in Table 1.

**Table 1:** Log *P* and extraction efficiency expressed as recoveries for six basic analytes extracted as a mixture using the microfluidic EME chip (mean ± SD, *n* = 3). Extractions were performed under standardized conditions (extraction potential of 1.0 V, flow rate of 2.0 µL min^−1^ in both channels). Log *P* values were obtained from PubChem [22, 28–32]

Compound	Log *P*	Recovery % ± RSD
Pethidine	2.46	45 (14)
Lidocaine	2.84	47 (13)
Haloperidol	3.66	50 (10)
Nortriptyline	4.43	46 (10)
Loperamide	4.77	38 (5)
Methadone	5.01	42 (12)

Lidocaine exhibited a recovery (47%) comparable to that obtained in the single-analyte extractions, demonstrating that efficient extraction is maintained in the presence of other analytes. The additional analytes had recoveries between 38 and 50%, with most exceeding 40%, indicating consistent extraction across the chosen analytes. Low and stable currents were observed throughout extractions, confirming a stable liquid membrane. Together, these results demonstrate that the microchip can be applied for simultaneous extraction of multiple basic analytes and is not limited to single-compound applications.

### Evaluation

Lidocaine was determined both in the acceptor and in the sample after extraction, and this enabled the establishment of a mass balance for the three-phase system. Very interestingly, from this mass balance experiment, no lidocaine was trapped in the liquid membrane. We explain this by low tortuosity and very rapid electrokinetic transfer across the liquid membrane. The linearity of the system was evaluated for lidocaine at relatively high concentrations in the range 5–25 µg mL^−1^ (see Fig. SI3 in Supporting information) to probe the limits of the current design. Linearity was obtained up to 20 µg mL^−1^ with an *R*^2^ value of 0.99. At 25 µg mL^−1^, deviation from linearity was observed, and this was explained by saturation of the liquid membrane.

The limit of detection (LOD) and limit of quantification (LOQ) were calculated from the calibration curve according to LOD = 3.3 *σ*/*S* and LOQ = 10 *σ*/*S*, where *σ* was the residual standard deviation of the regression and *S* was the slope of the calibration curve. The LOD and LOQ for lidocaine were determined to be 1.34 and 4.06 µg mL^−1^, respectively. Concentrations below the LOQ were included only for evaluation of linearity and were not used for quantitative determination. The analytical performance was demonstrated over a relatively high concentration range, which is consistent with the conceptual nature of this study and its focus on demonstrating feasibility. In addition, downstream analysis by CE-UV is less sensitive than LC-UV, which further explains the investigated concentration range.

The repeatability of the system was assessed for lidocaine at 20 µg mL^−1^. Six consecutive and independent extractions were performed. Between each extraction, the chip was fully disassembled from the chip holder and tubings, cleaned, and refilled. The repeatability was calculated to 16% RSD, and this was considered acceptable at this early and conceptual stage of development.

### Membrane replenishment and carryover

**Fig. 5:**
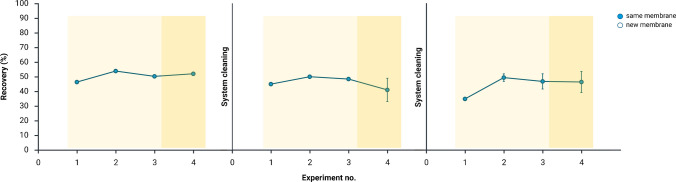
Extraction efficiency expressed as recovery over multiple consecutive extractions in the microchip (mean ± SD, *n* = 3, note: in some cases, error bars are smaller than the symbol size and therefore not visible). Each extraction consisted of three sequential extractions (experiments 1–3) collected at 10-min intervals under standardized conditions (extraction potential of 1.0 V, flow rate of 2.0 µL min^−1^ in both channels), followed by membrane replenishment and an additional extraction (experiment 4) performed under the same conditions. Light yellow shading and filled blue circles represent extracts obtained with the same membrane, whereas dark yellow shading and open blue circles represent extracts collected after introducing fresh liquid membrane without chip disassembly. Created in BioRender. Nguyen, A. (2026) https://BioRender.com/3bw85ea

Recovery data collected in Fig. 5 demonstrated that the system was stable for up to 60 min of operation with the same liquid membrane. For longer operations, replenishment of the liquid membrane was required. Fresh NPOE was easily introduced into the middle channel of the chip by pipetting. After 10 min of stabilization, the electric field was once again applied across the membrane. Extraction currents remained stable during the entire series of extraction, including after membrane replenishment (Fig. 5). The first extract after replenishment consistently had slightly lower recoveries compared to the subsequent extracts, which were similar (Fig. 5).

To evaluate the level of carryover between extractions, three blank extractions were performed using 10 mM HCl as both the test and acceptor solution, following an extraction of 20 µg mL^−1^ lidocaine. The calculated carryover (1%) was likely due to residual analyte retained inside the chip, the tubing, or the chip holder, despite flushing in between extractions. While the measured level was minor, it highlights the importance of thorough cleaning protocols or the use of fresh chips, tubing, and holders to eliminate analyte carryover between extractions.

### Comparison with existing micro-EME formats

To further clarify the analytical novelty of the microchip system and to position it relative to previously reported micro-electromembrane extraction (micro-EME) formats, a qualitative comparison with representative systems employing FLMs and SLMs is provided in Table 2. The comparison focuses on key design-related (fixed) and typical operational parameters (variable) rather than exhaustive analytical figures of merit, in line with the conceptual scope of this study.

**Table 2:** Qualitative comparison of representative micro-EME systems and the present microchip with a micro-pillar stabilized liquid membrane, highlighting key design features and typical operating conditions. Contact areas between membrane and donor/acceptor are approximate values calculated from reported device dimensions and are intended for qualitative comparison. Information for literature systems was obtained from [9, 33]

**Parameter**	**On-chip EME with FLM (Asl et al. **[33]**)**	**Micro-EME with FLM (Kubáň & Boček **[9]**)**	**Microchip with micro-pillar stabilized liquid membrane (this study)**
Platform/device type	PMMA chip with two channels separated by a porous membrane	PFA tubing micro-unit with liquid plugs	Thiol-ene microchip with three channels, separated by micro-pillars
Extraction mode	Three-phase EME	Three-phase EME	Three-phase EME
Membrane type	SLM	FLM	Micro-pillar-stabilized liquid membrane
Membrane thickness	Defined by porous support (200 µm)	Defined by liquid plug length (1.3–3.8 mm)	Defined by channel gap (180–420 µm)
Contact area between donor/acceptor and membrane	~ 30 mm^2^	~ 0.8 mm^2^	~ 0.38 mm^2^
Detection	HPLC–UV	UV–vis spectrophotometry and CE	CE-UV
Applied voltage/current	~ 40 V/µA	100 V/1–15 µA	1 V/0.1 µA
Flow rates	10–60 µL min^−1^	Stagnant	0.2–2 µL min^−1^
Extraction time	~ 30 min	5 min	10 min
Volume of liquid membrane	µL (impregnated)	1–3 µL	0.2 µL
Sample volume	1 mL	1.5 µL	20 µL
Acceptor volume	20 µL	1.5 µL	20 µL

Table 2 highlights several key differences between this system and previously reported micro-EME formats. In contrast to chip systems employing SLMs, the present design eliminates the need for porous support and membrane impregnation, thereby simplifying device preparation, enhancing chip reusability, and enabling direct control of the liquid membrane geometry. Compared to FLM-based micro-EME formats, in this case with liquid plugs in tubing [9], the present system offers a microchip format with a spatially confined membrane region that is well suited for microfluidic operation, integration with on-chip separation or detection, and enables reuse of the chip with different sample, acceptor, and liquid membrane compositions. In addition, the chip system enables stable extraction at markedly lower voltages than many reported micro-EME systems, which can be attributed to the short electrode spacing and chip-scale geometry. These design advantages are accompanied by certain limitations. The smaller interfacial area defined by the micro-pillar gaps likely contributes to the absence of preconcentration under the investigated conditions, consistent with the mass transfer limitations discussed earlier. Furthermore, extraction performance is sensitive to flow conditions and filling reproducibility, and assembly and handling are currently manual. At the same time, the absence of a porous support provides increased flexibility in liquid membrane selection between extractions, while membrane thickness and interfacial area can be optimized through microstructural design. Overall, this work introduces a first-generation microfluidic EME platform that enables renewable liquid membranes and low-voltage extraction and provides a foundation for further design exploration and application-oriented studies.

### Greenness

**Fig. 6:**
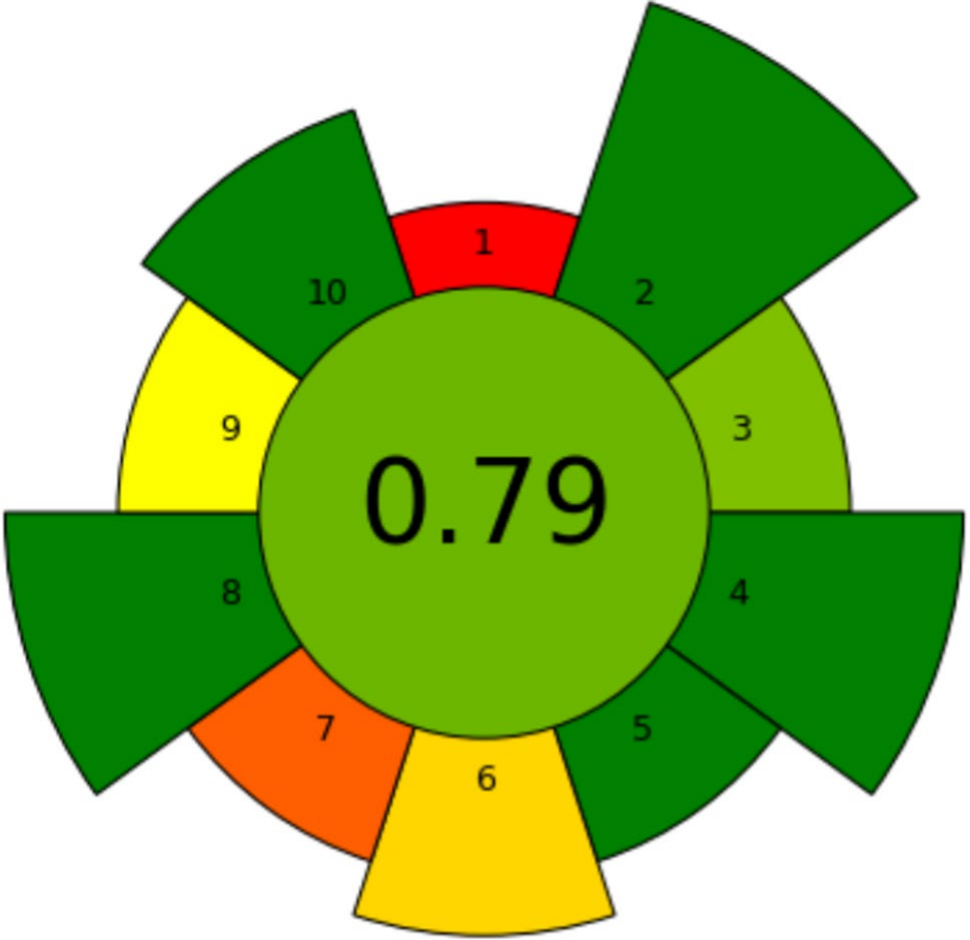
The AGREEprep greenness assessment score of 0.79 for the microchip system, evaluated based on ten weighted criteria (specified in SI1)

The greenness of the system was assessed using the AGREEprep metric tool [34]. The total AGREEprep assessment is summarized in Fig. 6, showing a final score of 0.79 (see SI1 in Supporting Information for detailed assessment). The greenness scores were high for criteria 2, 3, 4, 5, 8, and 10, and low for criteria 1, 6, 7, and 9. Extractions were carried out ex situ in the laboratory, and therefore, the score for criterion 1 was low. Criterion 6 received a low score due to the limited sample throughput (3 to 6 samples per hour). Additionally, the extractions were performed manually, contributing to the low score for criterion 7. Capillary electrophoresis was used for sample analysis, which influenced the score for criterion 9.

The AGREEprep score of 0.79 obtained in this study is high compared to previously reported EME systems [35–39]. This can be attributed to the miniaturized format, very low consumption of energy, sample, solvents, waste, and safe procedures. Although the whole process is currently operated in a manual, single-extraction mode, there is a potential for automation, which could enhance throughput and efficiency [23, 40]. The greenness evaluation confirms the system’s suitability for green sample preparation.

## Conclusion

This study introduced a microfluidic device for electromembrane extraction, where the liquid membrane was stabilized by rows of micro-pillars. The geometry provided a well-defined liquid–liquid interface and allowed easy membrane replenishment and reuse of the device. Fabricated in thiol-ene, the device offered an appropriate balance between hydrophilic and hydrophobic properties, ensuring compatibility with both aqueous and organic phases, as well as high chemical resistance and biocompatibility. Using lidocaine as a test substance, stable operation was demonstrated with recoveries up to 50%. The liquid membrane remained stable for at least 1 h of extraction and was easily replenished for extended operation. The microfluidic device presented in this paper represents a first-generation platform, and further analytical development and validation are required before routine application can be envisioned. Future studies should focus on additional design exploration and optimization, expanded analyte panels, and extractions from real samples. The findings and experiences reported in this article are important, as they establish a conceptual foundation for microfluidic EME with renewable liquid membranes and indicate potential for in-field sample preparation and future integration with on-chip detection.

## Supplementary Information

Below is the link to the electronic supplementary material.

Supplementary file1 (DOCX 282 KB)

## Data Availability

Data will be made available on request.
