# Supplementary material for: Microfluidic device for electromembrane extraction with a micro-pillar stabilized liquid membrane
Source: Anal Bioanal Chem. 2026 Mar 6;418(11):3251–62. doi: 10.1007/s00216-026-06427-z (PMC13197266; doi:10.1007/s00216-026-06427-z)
Supplement: Supplementary file 1 — Supplementary file1 (DOCX 282 KB) [file 216_2026_6427_MOESM1_ESM.docx]

Article name: Microfluidic device for electromembrane extraction with a micro-pillar stabilized liquid membrane

Journal name: Analytical and Bioanalytical Chemistry

Author names: Anna Thu Hoai Nguyen^a^, Nickolaj J. Petersen^a^, Stig Pedersen-Bjergaard^a,b^, Jörg P. Kutter^a*^

^a^ Department of Pharmacy, Faculty of Health and Medical Sciences, University of Copenhagen, Universitetsparken 2, 2100 Copenhagen, Denmark

^b^ Department of Pharmacy, Faculty of Mathematics and Natural Sciences, University of Oslo, P.O Box 1068 Blindern, 0316 Oslo, Norway

***** Corresponding author at: Department of Pharmacy, Faculty of Health and Medical Sciences, University of Copenhagen, Universitetsparken 2, 2100 Copenhagen, Denmark; Email: [jorg.kutter@sund.ku.dk](mailto:jorg.kutter@sund.ku.dk), telephone: +4535320399


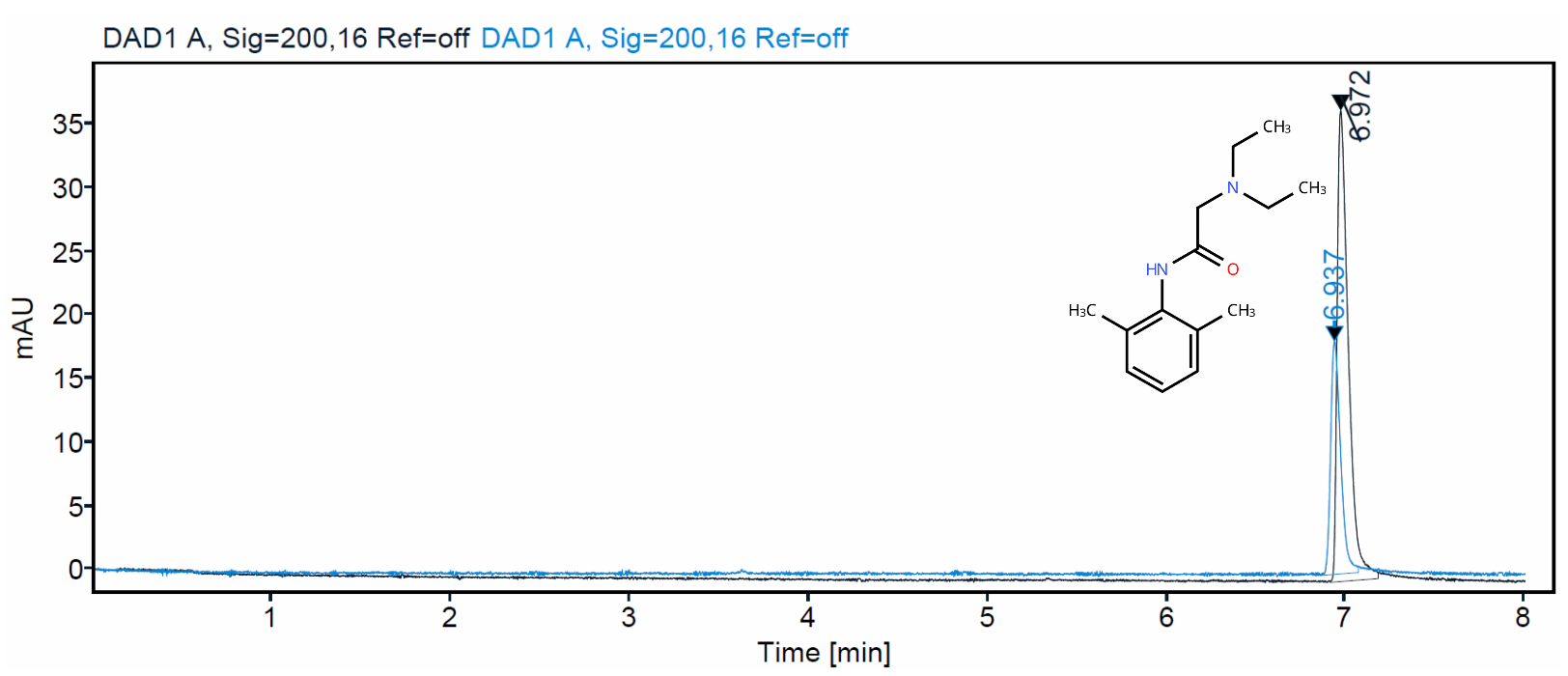


**Fig. SI1:** **CE electropherograms of lidocaine** in the test solution (standard; black trace) and acceptor (after extraction; blue trace) with migration times of 6.97 and 6.93, respectively. UV detection at 200 nm. The chemical structure of lidocaine is shown for reference.

**
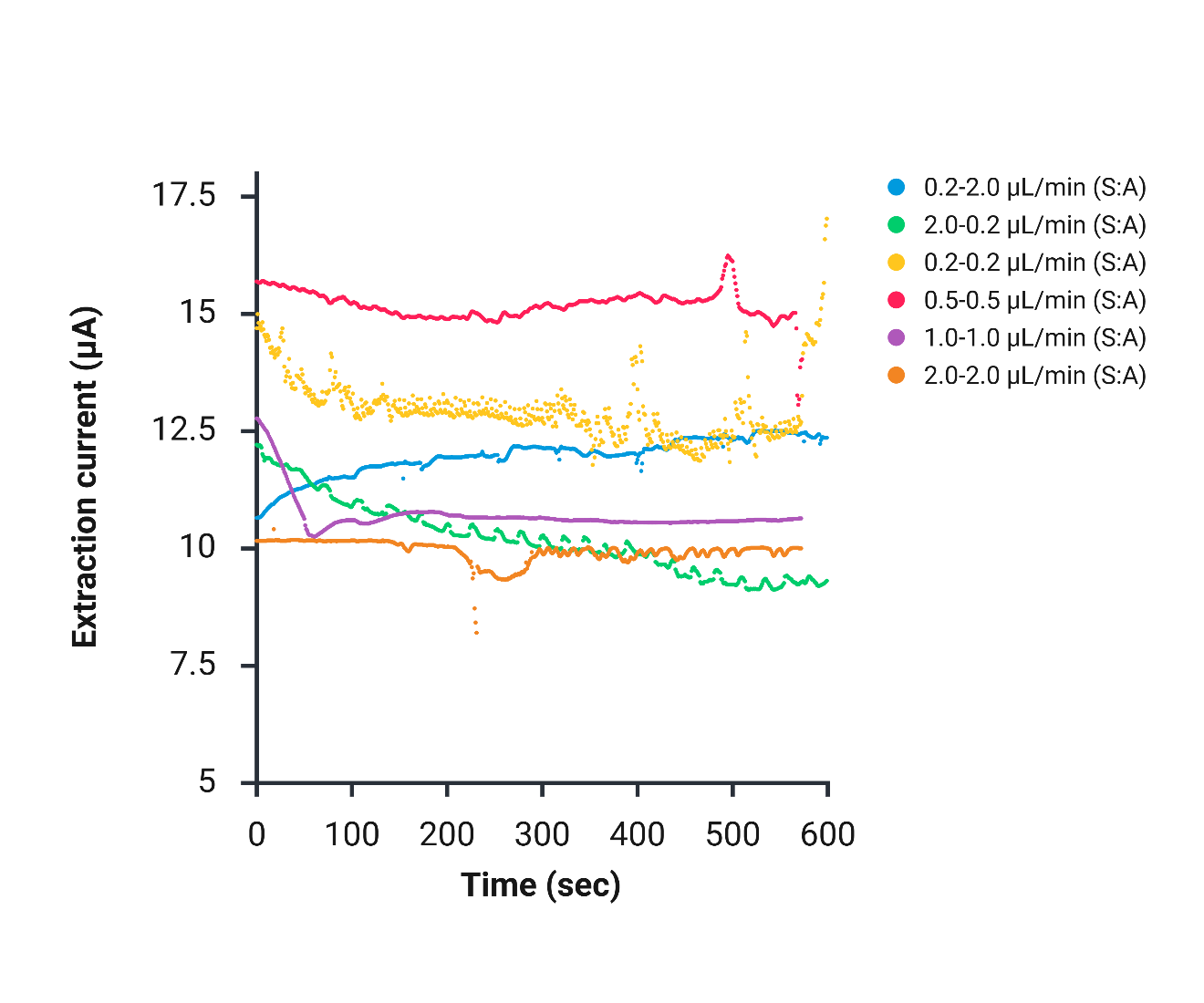
**

**Fig. S2: Extraction current (µA) over time during extraction in the microchip with different configurations of sample and acceptor flow rates (S:A)** and an applied extraction potential of 15 V. Stable current over time indicated an intact and stable liquid membrane. For all configurations, the current was considered stable (≈ 10-15 µA). Different colors correspond to; blue: flow 0.2-2.0 µL/min (S:A), green: flow 2.0-0.2 µL/min, yellow: 0.2-0.2 µL/min (S:A), red: 0.5-0.5 µL/min (S:A), purple: 1.0-1.0 µL/min (S:A), orange: 2.0-2.0 µL/min (S:A)


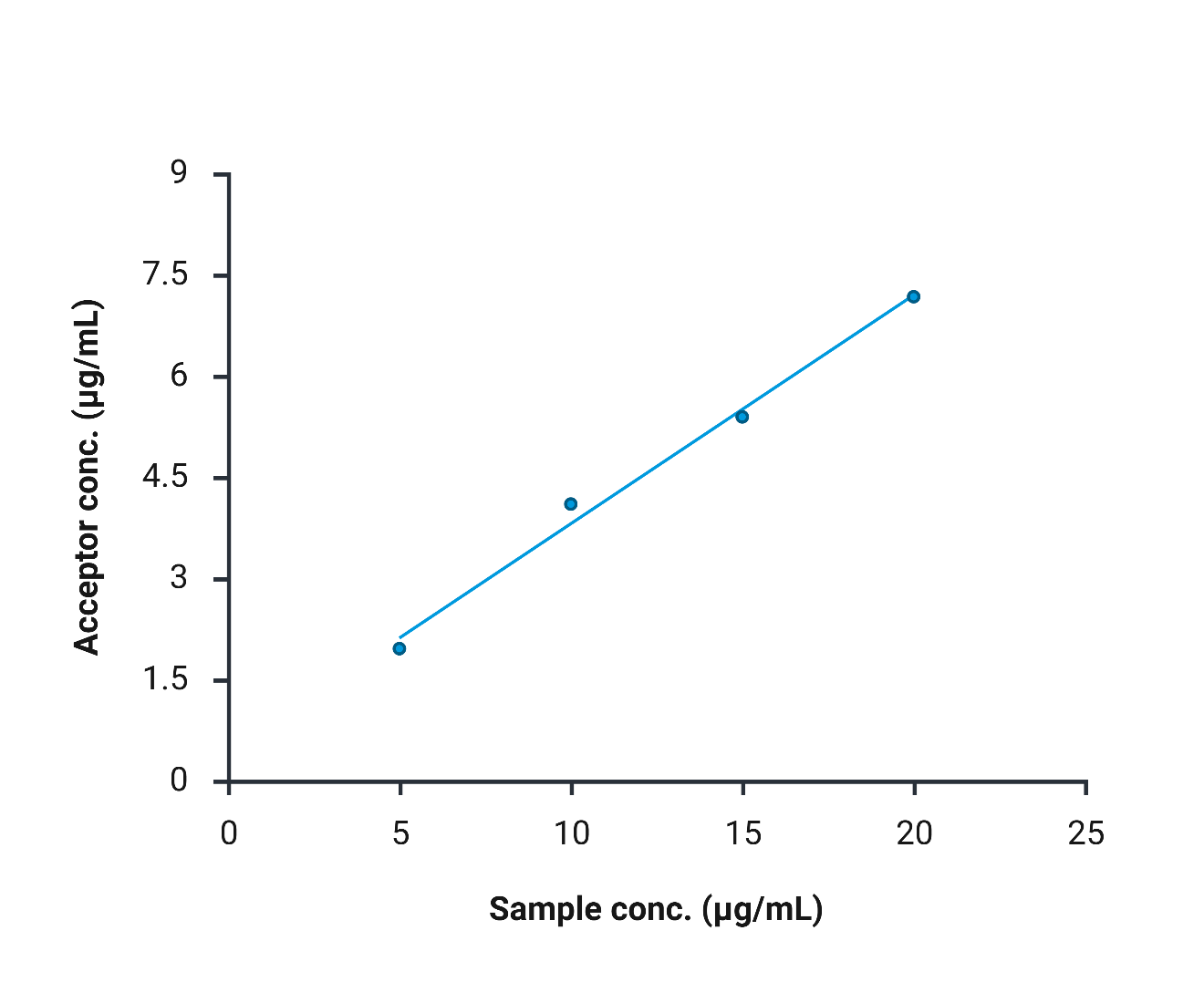


**Fig. S3: The linear response of lidocaine in the concentration range 5-20 µg/mL** extracted under standardized conditions (extraction potential of 1 V, flow rate of 2.0 µL/min in both channels). y=0.34x+0.42, R^2^=0.99

**S1. Greenness evaluation**

| Criterion | Criterion description | Weight |
| --- | --- | --- |
| 1 | Favor in situ sample preparation | 1 |
| 2 | Use safer solvents and reagents | 5 |
| 3 | Target sustainable, reusable, and renewable materials | 2 |
| 4 | Minimize waste | 4 |
| 5 | Minimize sample, chemical, and material amounts | 2 |
| 6 | Maximize sample throughput | 3 |
| 7 | Integrate steps and promote automation | 2 |
| 8 | Minimize energy consumption | 4 |
| 9 | Choose the greenest possible post-sample preparation configuration for analysis | 2 |
| 10 | Ensure safe procedures for the operator | 3 |

**Table SI1: Overview of the AGREEprep assessment criteria used to evaluate the greenness of the microchip system.** Each criterion is described briefly with its corresponding default weight (ranging from 0 to 5) toward the overall greenness score, reflecting the relative importance of each aspect in assessing analytical sample preparation greenness [28]

Criterion 1 favors in situ sample preparation, minimizing time, material, and energy. All the experiments were performed in the laboratory (ex situ), and the score was set to zero. Criterion 2 considers the use of toxic materials*.* The total volume (NPOE + HCL) was 10.4 µL. Because the total volume was less than 10 mg per sample, the score was set to 1.0. Criterion 3 addresses sustainable, and renewable materials as well as the reusability of these. In this study, the material was the microchip. Each chip could be reused for up to one month (up to 100 times), meaning >75% of materials are sustainable or renewable, scoring 0.75. Criterion 4 concerns waste. The waste per sample was: $1 \mu L organic solvent+20 \mu L sample size+chip 0.016 g=0.021 mL and 0.016g$, receiving a score of 1.0. Criterion 5 favors smaller sample sizes. In this case, the sample size was 20 µL, and the score was calculated to 1.23 ≈ 1.0. Criterion 6 evaluates throughput, which is determined by the overall duration of the sample preparation stage. Extraction times were either 10 or 20 minutes, so three to six samples were collected per hour, corresponding to a score of 0.42. Criterion 7 reflects procedural steps and automation. The system consisted of three steps (filling the chip, pipetting liquid membrane into system, transferring extracts into CE vials), receiving a score of 0.75. Because the system is fully manual, the final score is 0.19. Criterion 8 relates to power consumption per analysis. For each extraction, the power supply used 0.0006 W and the two syringe pumps used 25 W. The extraction time was 10-20 minutes, corresponding to 8 Wh, scoring 1.0. Criterion 9 relates to post-sample preparation configuration for analysis. All extracts were analyzed with CE, and the score was set to 0.5. The last criterion 10 covers safety. In this setup, no hazards or operator exposure were identified, setting the score to 1.0.
